# Gender determination from ridges using dermatoglyphics techniques

**DOI:** 10.6026/9732063002001008

**Published:** 2024-09-30

**Authors:** Manasi Patra, Niva Mahapatra, Shyam Sundar Behura, Lipsa Bhuyan, Girish MS, Samir PV, Pratik Surana

**Affiliations:** 1 Department of Oral & Maxillofacial Pathology, Kalinga Institute of Dental Sciences, KIIT Deemed To Be University, Bhubaneswar, Odisha; 2 Department of Pediatric and Preventive Dentistry, JSS dental College and Hospital, Affiliate to JSS Academy of Higher Education and Research, Mysuru, Karnataka; 3 Department of Pedodontics and Preventive Dentistry, Kalinga Institute of Dental Sciences, KIIT Deemed To Be University, Bhubaneswar, Odisha; 4 Department of Pedodontics and Preventive Dentistry, Maitri College of Dentistry and Research Centre, Durg, Chhattisgarh, India

**Keywords:** Dermatoglyphics, fingerprint pattern, forensic medicine

## Abstract

Fingerprint analysis for gender determination in Ganjam District, Southern Odisha is of interest in forensic science. Dermatoglyphics, the study of fingerprint patterns, leverages their genetic uniqueness and stability to identify individuals in forensic and disaster scenarios. Left thumb prints were collected and analyzed under magnification, followed by statistical evaluation. Results showed significant differences in fingerprint patterns between genders, confirming the method's accuracy. Thus, fingerprint pattern analysis is a valuable tool for gender identification in forensic medicine, criminal investigations, and during mass disasters.

## Background:

Forensic odontology is that branch of forensic medicine deals with the proper handling and examination of dental evidence and with the proper evaluation and presentation of the dental findings as defined by Keiser- Neilson [1]. Forensic dentistry has the following applications: Diagnostic, therapeutic examination and evaluation of injuries pertaining to jaws, hard tooth structure and soft tissue of oral cavity, Identification of a person in post-mortem cases as a part of criminal investigation procedure or in mass casualties, Identification, inspection and assessment of bite marks in sex crimes and child abuse cases [2]. Forensic dentistry includes numerous techniques such as: anthropometry, cheiloscopy, rugoscopy, dermatoglyphics, DNA analysis for human identification like gender determination, estimation of age, *etc.,* Dermatoglyphics is the study of fingerprints for determination of the gender of the individual, which is the least invasive and also a cost-effective procedure [3]. The term "fingerprint" predominately refers to an impression of the epidermal ridges of the fleshy distal portion of the finger. The study of fingerprints is called Dermatoglyphics [4]. Cummins and Mildo were credited for introducing the term dermatoglyphics. It is the study of the dermal ridge configuration of digits, palms and soles. Unlike most other physical characteristics, fingerprint patterns form very early in the womb, between 6 to 13 weeks of gestation. This means they are established before any environmental influences can be hold, making them even more unique [5]. Fingerprints are classified into broad categories like loops, whorls, and arches as proposed by Michael Kucken in 2005 [6]. But the true level of uniqueness lies within the miniature- the tiny details like ridge endings, bifurcations (where a ridge splits) and dots. These miniatures can number in the hundreds and vary greatly between individuals [7]. Fingerprint analysis is a cornerstone of identification, its intricate swirls and loops acting as a unique code for each individual, aiding in criminal investigations and security measures. Dermatoglyphic traits can vary across populations and are determined by genetics. Hence, they do not change with aging [8, 9, 10]. Studying these variations helps anthropologists understand human evolution, migration patterns and the relationships between different populations. But what if these ridges whispered more than just identity? Recent research in dermatoglyphics, the scientific exploration of skin ridges, suggests a fascinating possibility; a potential link between fingerprint pattern and gender. This article delves into this exciting frontier, exploring the specific ridge formations associated with masculinity and femininity, analyzing the scientific research that underpins these connections. Therefore, it is of interest to analyse the fingerprints of males and females in order to correlate the above fingerprints for determination of gender.

## Materials and Methods:

The sample size comprised of 205 subjects, 103 males and 102 females from Ganjam District, Odisha aged between 20 to 50 years. The participants were explained about the procedure and their informed consent was obtained for the study. The materials required for the study were blue ink stamp pad, white paper sheet and a magnifying lens.

## Methodology:

Each subject was instructed to wash their hands prior to recording their fingerprints to remove oil, dirt and sweat. The subjects were directed to dry their fingertips with a napkin and then the blue ink of the stamp pad was applied on the left thumb. The imprint of the fleshy distal portion of left thumb was recorded on a white chart paper and it was visualized under a magnifying lens.

## Inclusion Criteria:

All healthy subjects with intact left thumb irrespective of habits were included in the study.

## Exclusion Criteria:

Subjects with pathology of dermis, amputated left thumb or fingertip, burn injury / cuts/ scar of the fingertip of left thumb were excluded from the study.

## Evaluation:

**Figure 1:**
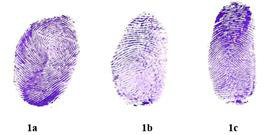
Type of figure print pattern

Fingerprints were recorded based on the criteria by Michael Kuckenas [6] which is as follows; (Figure 1a, Figure 1b, Figure 1c)

[1] Type 1 Whorl pattern: These are formed by any random ridges that encircle a core with two or more triradii.

[2] Type 2 Loop pattern: This pattern has numerous ridges that enter the finger tip from one side and leave from the same side.

[3] Type 3 Arch pattern: These are usually composed of ridges that cross from one side of fingertip to the other fingertip. It has no triadius.

## Statistical analysis:

Microsoft Excel 2016 was used to fabricate the data sheet. IBM SPSS Corp. in Armonk, New York for Windows, Version 25.0, was used for the statistical analysis. The data was normally distributed. Chi square statistics were applied to calculate the inferential statistics between the different variables. The statistical constant was fixed at p<0.05.

## Results:

The mean age of the study distribution was 41.74±13.811. There were 103 males and 102 females.

## Distribution of pattern of finger prints in the 205 sample population:

The commonest type of fingerprint which was observed in the present study was Type 1 (41.0%) followed by Type 2 (33.7%) and Type 3 (25.4%). There was a statistically significant difference between the types of fingerprint (p=0.023). The same has been graphically represented in Table 1.

## Distribution of finger print pattern commonly found in males and females:

It was seen that Type 1 fingerprint was the commonest among male participants and type 2 fingerprint was commonest for the female participants and it was statistically significant(p=0.001). Type 3 had a similar presentation in both the gender groups. There was a statistically significant difference between the presentations in both groups. The same has been graphically represented in Table 2. Linear logistic statistics was computed to calculate the significance of the distribution pattern and it was seen that a statistically significant correlation was observed between the gender and the type of finger pattern (p=0.002)

## Discussion:

Establishment of the identity of an individual is known as personal identification. Personal identification is very essential in cases of natural calamity like earthquakes, floods, cyclones, landslides *etc.* and also as a part of criminal investigation into mass murder, terrorist bombings, decomposed dead bodies or dismembered bodies in murder to hide the identity of an individual. Personal identification is a crucial component of forensic anthropology that includes the big fours *i.e.* age, sex, stature and ethnicity [11]. Various methods have been identified for determination of gender of an individual that include visual method (such as canine dimorphism, mandibular canine index *etc.*), microscopic methods using barr bodies, advanced method includes amplification of DNA extracted from dental pulp using PCR, study of enamel proteins AMEL, soft tissue methods like rugoscopy, cheiloscopy, dermatoglyphics etc [12]. The ridges of the finger are determined by multiple genes that have vital genetic information about an individual. Dermatoglyphics was first used for identification by Sir Edward Henry in 1893 [13]. Later, studies were conducted by many authors utilizing finger print as a tool for gender determination [8, 9, 14] In the above context, we too applied the procedure of fingerprint analysis in gender identification in a total of 205 subject' native to Southern Odisha, Ganjam District.

We found a statistically significant result among the entire 205 subjects, the commonest finger print was whorled pattern (type 1) followed by loop pattern (type 2) and least common variant was Arched pattern (type 3). [5, 15] In males, the most common fingerprint was a whorled pattern and in females the commonest finger print was a loop pattern and the difference was statistically significant in accordance with other studies. But the arch pattern was least common, equally distributed among the males and females. Linear logistics was also used and a statistical correlation was found between gender and the distribution pattern of the finger print. Numerous theories by many authors are proposed for finger print pattern formation. The first theory proposed by Kollmann stated that the pattern seen in finger prints is mostly due to a folding process caused by differential growth. Later bonnevie stated that there is formation of cylindrical cells in the basal layer of epidermis due to intense cell proliferation followed by formation of primary ridges due to the separation of epidermis and dermis. This gives the unique characteristics of the finger print pattern of a person and even in skin injury after regeneration, the finger print pattern remains unchanged throughout life. Another theory was proposed by Dell Munger regarding the formation of finger print pattern. They stated the primary ridge formation is mostly due to innervation of hexagonal pattern of axons. But later this theory was rejected by Monohunfola as the ridges in the finger are formed in the absence of innervations. Hence, due to the developmental process of formation of the fingerprint pattern, it is unique to only one individual and remains unchanged throughout life [9, 16]. In the recent times dermatoglyphic studies are not restricted to gender identification only but also has wide array of applications. Alterations in finger print pattern are also indicative of genetic disorders or syndromes, such as

[1] In Down syndrome, there is formation of radial loop in 4 and 5 digits.

[2] In Klein filter syndrome, the height of the axial tri-radius is accentuated in hypothenar pattern.

[3] In Rubinstein Taybi syndrome, four or more arches are found in the fingertips.

[4] Infants with cleft lip and palate have formation of numerous arches and loops in the digits and there is escalation in the triradii count [13].

Abnormalities in dermatoglyphics are also implicated in dental caries where the subjects exhibit rise in whorl pattern and the count of finger ridge pattern was elevated. Individuals with pre malignancy like oral Submucous fibrosis have a marked increase in arch pattern in their digits and a reduced whorl pattern. Thus, suggestive that study of dermatoglyphics can not only determine the gender of the person but can also give us an insight into various disorders [17]. However, there are limitations in fingerprint analysis with persons suffering from gross malformation of limbs. Recording the pattern of the digits is also tricky as an increase in the application of ink will result in improper prints [18].

## Conclusion:

There are numerous studies on dermatoglyphics aiding in the determination of gender. But our study is unique as it suggests a significant comparison between the gender and the type of fingerprint in Ganjam district of Southern Odisha. Hence, dermatoglyphics is useful in determination of gender in forensic sciences and could also be employed as critical evidence in criminal investigation.

## Figures and Tables

**Table 1:** Distribution of pattern of finger print in the 205 sample population

	**Frequency**	**Percent**	**Chi square**	**P Value**
Type 1	84	41	7.502	0.023*
Type 2	69	33.7		
Type 3	52	25.4		
Total	205	100		

**Table 2:** Distribution of finger print pattern commonly found in males and females

	**Male**		**Female**		**Chi Square**	**P Value**
	Frequency	Percent	Frequency	Percent		
Type 1	60	57.1	24	24	15.429	<0.0001*
Type 2	19	18.1	50	50	13.928	<0.0001*
Type 3	26	24.8	26	26	0	1
Total	105	100	100	100		
Chi Square	27.486		12.56			
P Value	<0.0001*		0.002*			
*statistically significant
